# Overtriage and undertriage in a prehospital system over 7 years

**DOI:** 10.1186/cc12214

**Published:** 2013-03-19

**Authors:** L Carenzo, F Barra, A Messina, D Colombo, T Fontana, F Della Corte

**Affiliations:** 1 Universitè del Piemonte Orientale A. Avogadro, Novara, Italy

## Introduction

The Novara 118 emergency medical system (EMS) dispatch center manages medical emergency calls coming from a region that spreads out over 1,400 km^2 ^and includes 88 towns and a population of 385,000 people; inhabitant density is 275 inhabitants/km^2^.

## Methods

Data collection from 1 January 2005 to 31 December 2011 was obtained (EMS software SaveOnLine^® ^Suite 4; REGOLA s.r.l., Turin, Italy). Triage used was the Medical Priority Dispatch System and severity was defined by color codes.

## Results

During the study period the Novara 118 dispatch center managed a total of 122,384 EMS interventions. Median (interquartile) overestimation (regardless of severity) was 10.0% (2.4 to 14.1%) while median underestimation was 1.5% (0.8 to 2.5%). See Tables 1 and 2.

## Conclusion

Overall there were no statistical differences between the observation years either for overtriage or undertriage. When observed individually, trauma showed the only significant overtriage reduction over time; there were no individual modifications in undertriage over time.

**Table 1:** Overtriage (%)

	Overtriage						
	
	2005	2006	2007	2008	2009	2010	2011
Other	3.03	2.47	5.30	4.95	4.06	4.58	4.17
Cardiac	13.23	15.30	27.06	23.72	19.28	19.17	20.14
Drunk	0.00	0.00	0.00	0.00	0.00	0.00	0.00
Poisoning	10.19	18.92	17.91	8.47	8.85	15.20	11.81
Oncological	0.00	2.78	1.05	1.03	3.03	2.47	0.00
Neurologic	9.97	10.05	15.59	15.18	14.57	13.32	13.32
Not identified	13.03	13.15	11.84	11.07	10.33	11.79	7.23
Mental	1.30	0.41	2.09	2.34	2.30	1.27	2.35
Respiratory	10.67	10.89	22.09	18.63	16.29	18.23	14.12
Trauma	12.97	12.68	10.42	8.59	6.78	7.74	7.56

**Table 2:** Undertriage (%)

			Undertriage				
	
	2005	2006	2007	2008	2009	2010	2011
Other	1.01	0.77	2.87	1.66	1.44	1.48	1.31
Cardiac	2.16	1.44	1.64	1.60	2.15	1.60	1.14
Drunk	0.00	1.89	0.00	0.00	0.00	0.00	0.00
Poisoning	8.33	0.00	0.00	1.69	0.00	1.60	0.00
Oncological	95.74	0.56	0.00	1.03	3.03	2.47	0.00
Neurologic	2.42	2.32	2.96	3.67	3.05	1.40	2.46
Not identified	1.72	2.13	1.87	2.20	2.04	2.74	3.50
Mental	1.74	0.83	1.05	1.17	0.00	0.42	0.39
Respiratory	2.30	4.16	3.76	3.75	2.57	2.72	2.46
Trauma	1.07	0.92	1.50	1.41	1.36	1.31	1.46

## References

[B1] Neely KW Eldurkar JA Drake ME Acad Emerg Med 2000 7 174 180 10.1111/j.1553-2712.2000.tb00523.x 10691077

